# Developing 400 MPa grade biodegradable Zn alloys with superior osteogenic and antibacterial performance

**DOI:** 10.1038/s41598-025-32236-y

**Published:** 2025-12-13

**Authors:** Jiawei Cai, Qiang Li, Shikai Li, Yan Chengyue, Zhe Liu, Zhiyong Zhang, Bin Cheng

**Affiliations:** 1 Translational Research Centre of Regenerative Medicine and 3D Printing, Guangdong Province Engineering Research Center for Biomedical Engineering, State Key Laboratory of Respiratory Disease, Department of Orthopaedic Surgery, Guangzhou Key Laboratory of Spine Disease Prevention and Treatment, Guangdong Provincial Key Laboratory of Major Obstetric Diseases, Guangdong Provincial Clinical Research Center for Obstetrics and Gynecology, The Third Affiliated Hospital, Guangzhou Medical University, Guangzhou, 510150 Guangdong P.R. China; 2 The Second Orthopedic Department of Zhangzhou Zhengxing Hospital, Zhangzhou, 363000 Fujian P.R. China

**Keywords:** Zinc alloys, Equal channel angular pressing, Mechanical properties, Osteogenesis, Engineering, Materials science

## Abstract

Biodegradable zinc (Zn) has emerged as a promising orthopedic implant material, capable of supporting bone repair while gradually resorbing in the body. Yet, its relatively low strength has restricted its use in high load-bearing scenarios. The addition of lithium (Li) improves mechanical strengths of Zn alloys, of which are comparable to that of pure Ti. Here, we present a Zn-0.8Li alloy system enhanced through alloying and equal channel angular pressing (ECAP). By tuning the processing temperature, the alloys attained superior mechanical performance, with tensile strength reaching 434 MPa and elongation of 65% at 200 °C. The dominant strengthening mechanisms were identified as grain boundary strengthening and dislocation strengthening. Corrosion assessment revealed a stable degradation rate of ~ 5.5 μm/year after 30 days of immersion, with localized attack at second phases and grain boundary corrosion in ultrafine-grained microstructures (T-150 and T-200 samples), whereas a fine-grained microstructure (T-300 sample) exhibited suppressed boundary corrosion. In vitro studies confirmed excellent cytocompatibility and osteogenic potential of the ECAP-treated Zn-0.8Li alloys compared with bioinert Ti. Furthermore, antibacterial tests demonstrated inhibition rates exceeding 90% against *E. coli* colonies.

## Introduction

Zinc (Zn) has recently attracted growing interest as a biodegradable metal for temporary implants, owing to its moderate corrosion rate and favorable biocompatibility^1,2^. For example, a vascular stent that made from Zn-0.1Li alloys shows lower penetration rates (2.93 μm/year) than that of pure Zn (27 μm/year) after implantation for 12 months^3,4^. The limitation is the poor mechanical properties of pure Zn that has strength below 200 MPa after thermomechanical process^5^. As an orthopedic internal fixation device, the strength should be over 300 MPa for providing adequate support^6^. Thus, it is necessary to improve mechanical properties of pure Zn by combining alloying elements and thermomechanical process. Among alloying strategies, lithium (Li) has been identified as a particularly effective strengthening element for Zn-based systems, making Zn-Li alloys promising candidates for load-bearing medical devices^7–12^. *Zhao et al.*. reported that in hot-rolled Zn-xLi alloys (x = 0.2–0.7 wt%), Li contents above 0.4 wt% reduced ductility^13^. In contrast, *Li et al.*. later demonstrated that a hot-warm rolled Zn-0.8 wt.%Li alloy exhibited remarkable strength combined with an elongation-to-failure of nearly 80%, which they attributed to sub-micron grain formation^14^. Moreover, Zn-0.8Li based alloys show the improved osteogenic properties compared with Ti, which is attributed to the activation of a bone-repaired related PI3K-AKT signaling pathway^11^.

Equal channel angular pressing (ECAP) has emerged as an effective severe plastic deformation method for refining metallic microstructures and improving strength without sacrificing ductility^15^. For instance, *Ye et al.*. produced Zn-0.1Mg alloys via ECAP with an average grain size of ~ 1 μm, achieving a tensile strength of 383 MPa and elongation of 45.6%^16^. Lower ECAP temperatures further enhanced grain refinement, yielding superior combinations of strength and ductility through activation of non-basal slip systems. Similarly, Ren et al. found that ECAP processing at room temperature increased the elongation of Zn-1Cu alloys from 25.9% to 94.2%^17^. *Bednarczyk et al.*. also confirmed that Zn-Cu/Mn/Ag alloys exhibited greatly improved ductility due to dynamic recrystallization (DRX) coupled with fine particle pinning during ECAP^18^. Despite these advances, relatively few studies have focused on the role of ECAP in tuning the microstructure and mechanical response of Zn–Li alloys^19,20^. According to the binary Zn-0.8Li phase diagram, temperature determines the phase components in Zn-0.8Li alloys^21^. Since ECAP parameters—particularly temperature—critically affect grain refinement and defect evolution in metals^17^, a systematic investigation of their impact on Zn-0.8Li alloys is essential. To advance biodegradable Zn alloys toward clinical use, it is necessary to clarify how ECAP temperature governs microstructural development, mechanical performance, corrosion resistance, and biological response. Such a combined microstructure-property-biology map for Zn-0.8Li alloys through altering ECAP temperature has not been reported previously.

**Scheme 1:**
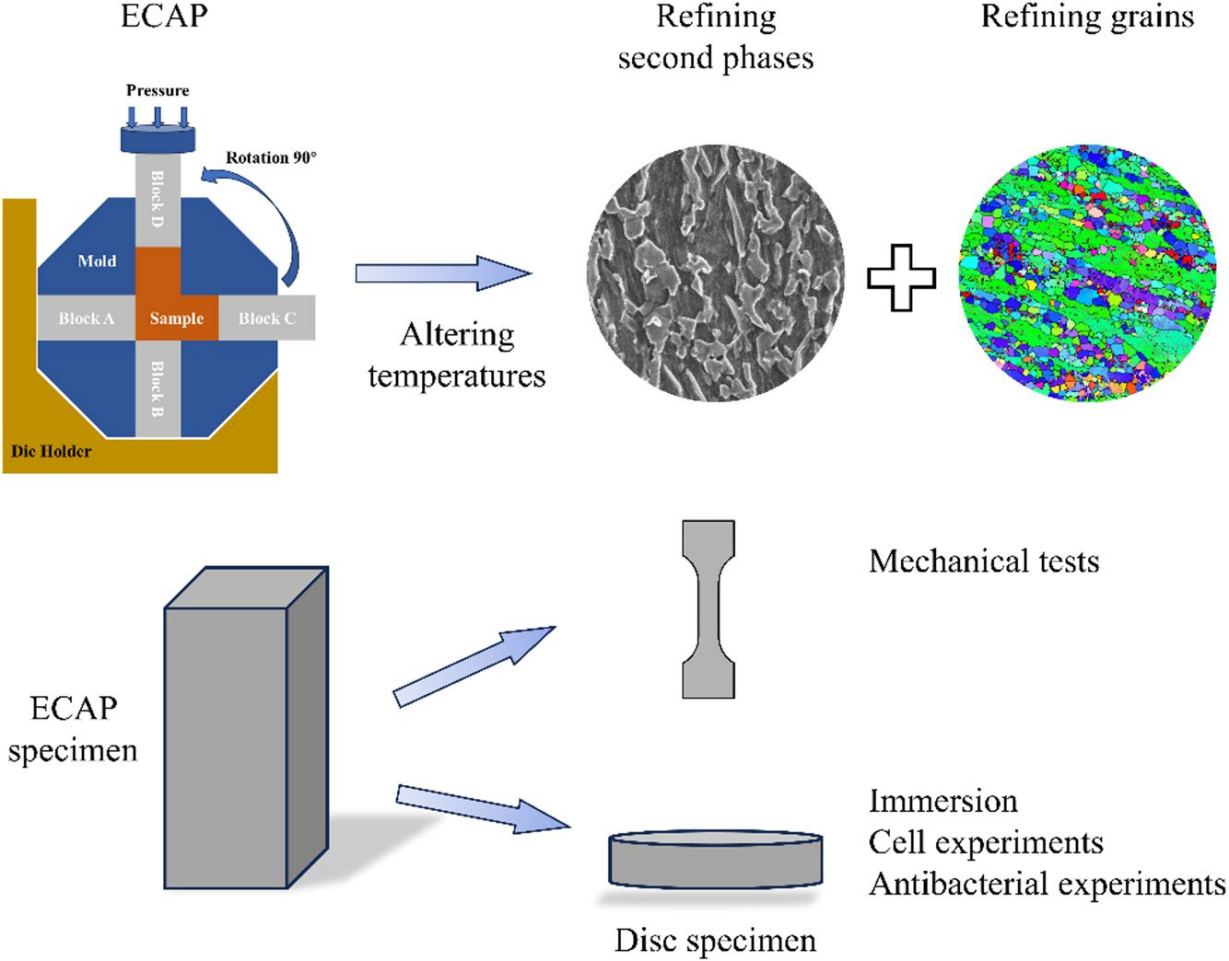
Schematic illustration of the preparation of high-strength Zn-0.8Li alloys after ECAP and characterizations.

In this work, ultrafine-grained Zn-0.8Li alloys were fabricated by ECAP, and their microstructure, mechanical properties, corrosion behavior, cytocompatibility, osteogenic potential, and antibacterial activity were comprehensively evaluated, as shown in Scheme 1. These findings not only enrich current understanding of Zn-based biodegradable metals but also highlight their potential as high-performance candidates for orthopedic implant applications.

## Experimental procedures

The as-cast Zn-0.8Li alloy ingots used in this study were provided by the Hunan Institute of Rare Earth Metal Materials. The chemical composition was determined by inductively coupled plasma atomic emission spectroscopy (ICP-AES, iCAP6300, USA), confirming that the actual Li content was 0.79 wt%, as shown in Table 1.

**Table 1:** Chemical compositions of as-cast Zn-0.8Li alloy.

Sample	Weight fraction, wt%					
	Li	Fe	Ni	Al	Si	Zn
Zn-0.8Li	0.79	0.0022	0.0029	0.0017	0.0011	Basal

Rectangular bars (10 × 10 × 90 mm) were sectioned from the ingots and subjected to equal-channel angular pressing (ECAP). The ECAP route followed the procedure previously described in the literature^22,23^. Processing was performed at 150 °C, 200 °C, and 300 °C, denoted as T-150, T-200, and T-300, respectively. Each specimen underwent 12 passes, with a 5-minute holding period after every four passes. Post-deformation, plates of 10 × 10 × 2 mm were extracted for systematic evaluations, including microstructural analysis, electrochemical and immersion assays, cellular tests, osteogenic induction, and antibacterial performance.

To investigate the microstructures of the ECAP-processed alloys, multiple characterization methods were employed. Optical microscopy (OM, ZEISS Axio Lab A1, Germany), scanning electron microscopy (SEM, ThermoScientific, USA), and electron backscatter diffraction (EBSD, Bruker, Germany) were used to examine grain morphology and crystallographic orientation. The elemental distribution was mapped by energy-dispersive spectroscopy (EDS, Oxford, UK) integrated with SEM. Samples were prepared by sequential grinding, polishing, and etching, using a 4 wt% nitric acid–alcohol solution as the etchant. For EBSD samples, the mechanically polished samples need to be electrochemically polished. The electrolytic polishing solution is composed of 50 vol.%H_3_PO_4_ and 50 vol.%C_2_H_5_OH solution, and the electrically polished process of the specimen is conducted at 15 V and − 30 ℃ for 10–15 s to yield clean surface. Mechanical properties were evaluated by tensile testing with a universal testing machine (Suns UTM4294X, China) at a strain rate of 10⁻³ s⁻¹, using dog-bone specimens of 2 × 2 × 7.5 mm tested at room temperature. Three tensile samples were tested to obtain average values and standard deviations.

Electrochemical behavior was characterized in a three-electrode configuration on an electrochemical workstation (CHI660C, Chenhua, China). A platinum electrode as counter electrode (CE), a saturated calomel electrode (SCE) as reference electrode (RE), and samples being tested as working electrodes (WE). The open-circuit potential was first monitored for 3600 s, followed by potentiodynamic polarization scans ranging from − 1.4 V to − 0.8 V at a sweep rate of 0.1 mV·s⁻¹. For immersion tests, specimens were placed in Hank’s solution for 7 and 30 days. The ratio of the area of ECAP samples and the volume of Hank’s solution is 20 mL/cm^2^ during immersion tests according to the ASTM G31 standard. The morphology of the corrosion products was examined by SEM. After immersion, samples were rinsed with distilled water, dried, and then cleaned in 200 g/L chromium trioxide (CrO₃) solution to remove corrosion layers. Three samples without corrosion products were weighed to calculate the corrosion rates according to the equation: $\:{v}_{corr}=\frac{\varDelta\:m}{\rho\:At}$. Where $\:\varDelta\:m$ is weight loss, ρ is density, A is the exposed area, t is immersion time. The underlying alloy surfaces were subsequently re-examined by SEM to reveal corrosion patterns.

In vitro cytotoxicity and cell-based assays were performed using RAW264.7 and MC3T3-E1 cells. RAW264.7 and MC3T3-E1 cells were seeded in 96-well plates at a density of 3 × 10^4^ cells per well. Both cell lines were maintained in α-MEM supplemented with 10% fetal bovine serum (FBS) and 1% penicillin–streptomycin, under standard culture conditions (37 °C, 95% relative humidity, and 5% CO₂). The medium was refreshed every 48 h. Prior to use, alloy specimens were ultrasonically cleaned and sterilized under UV light for at least 4 h. Sterilized samples were then immersed in FBS-enriched culture medium at a ratio of 1.25 mL/cm² for 24 h according to the ISO 10993-5 standard. The extracts were collected, centrifuged, and stored at 4 °C to ensure sterility and integrity for subsequent tests.

For cytotoxicity evaluation, RAW264.7 and MC3T3-E1 cells were harvested by trypsinization, resuspended, and exposed to either undiluted or 50% diluted extracts for 72 h. Cell viability was quantified by the Cell Counting Kit-8 (CCK-8, Dojindo, Japan) assay, using the formula:

$$\:Cell\:viability=\frac{{OD}_{e}-{OD}_{b}}{{OD}_{c}-{OD}_{b}}\times\:100\%$$

where $\:{OD}_{e}$ represents absorbance from cells cultured with extracts, $\:{OD}_{b}$ denotes the blank control (medium without cells), and $\:{OD}_{b}$ corresponds to the untreated control. Post-treatment, cells were stained with Calcein-AM/PI and imaged under an inverted fluorescence microscope (Carl Zeiss Axio Vert.A1, Germany) to evaluate morphology and viability.

For osteogenic assays, MC3T3-E1 cells were seeded in osteoinductive medium prepared with 50% extracts. Alkaline phosphatase (ALP) staining was conducted after 7 and 14 days following the manufacturer’s protocol, with medium replaced every 48 h. Mineralized matrix deposition was analyzed by Alizarin Red S staining (ARS). After 14 days of induction, cells were fixed with 3.7% formaldehyde, rinsed with PBS, and stained with 40 mM ARS (pH 4.1). This experimental design enabled an integrated assessment of cytocompatibility and osteogenic potential of alloy-derived extracts on osteoblast precursor cells.

Alloy extracts were prepared in accordance with ISO 10993-12:2012. The antibacterial performance against *Staphylococcus aureus* (S. aureus) and *Escherichia coli* (E. coli) was evaluated using the spread plate technique. Each Zn alloy was tested in triplicate, while pure Ti served as the reference material. Bacterial strains, revived from frozen stocks, were grown overnight in Luria–Bertani (LB) broth at 37 °C and 150 rpm, followed by 3–5 passages on LB agar. Actively growing cultures were then diluted with sterile phosphate-buffered saline (PBS) to 10⁸ CFU·mL⁻¹. Sterilized alloy and Ti discs (10 mm diameter, 1 mm thickness) were placed in 24-well plates, each inoculated with 1 mL of bacterial suspension. After incubation at 37 °C for 24 h, LB agar plates were prepared by coating with a 100-fold diluted PBS bacterial suspension. The antibacterial rate (AR) was calculated using the equation:

$$\:AR\left(\%\right)=\frac{{N}_{c}-{N}_{s}}{{N}_{c}}\times\:100\%$$

Where $\:{N}_{c}$ represents the average colony count on Ti, and $\:{N}_{s}$ corresponds to that on Zn alloys. According to SN/T 2399 − 2010, materials achieving an AR ≥ 90% were defined as antibacterial.

Results were expressed as mean ± standard deviation. Statistical differences among groups were analyzed using one-way ANOVA followed by Tukey’s post hoc test (GraphPad Prism v6.1, GraphPad Software, San Diego, USA). A *P* value < 0.05 was considered statistically significant.

## Results

### Microstructures

**Fig. 1:**
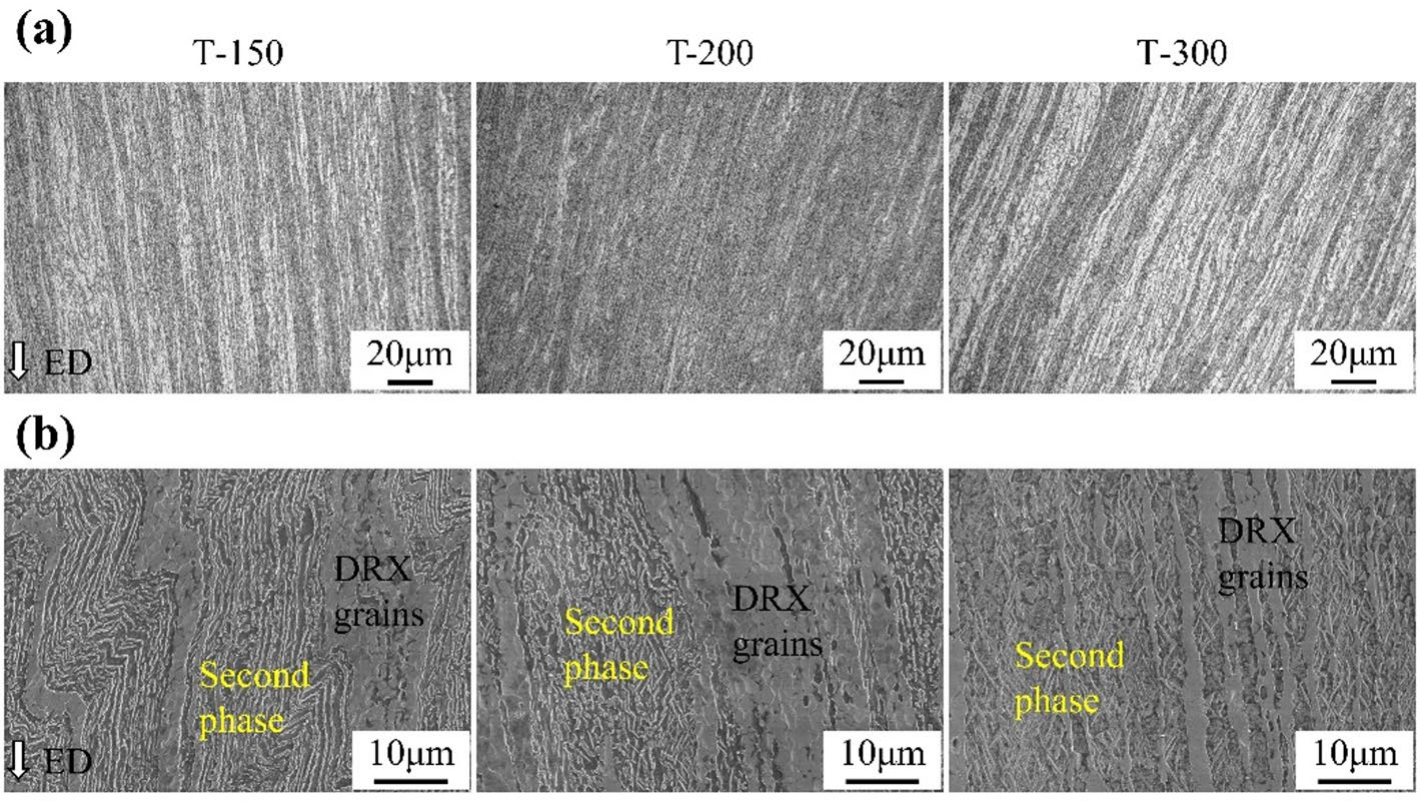
Microstructures of Zn-0.8Li alloys after ECAP at different temperature. **(a)** OM images. **(b)** SEM images. (ED is ECAP direction).

The microstructures of ECAP-processed Zn-0.8Li alloys were examined by OM (Fig. 1a). The Zn matrix and secondary phases were elongated along the extrusion direction (ED), forming an alternating pattern. Representative SEM images are shown in Fig. 1b. After deformation, both dynamically recrystallized (DRX) grains and elongated secondary phases were present, appearing as fine granular structures and strip-like features aligned with ED. As the ECAP temperature increased, the secondary phases became thinner, accompanied by Zn grains with higher aspect ratios.

**Fig. 2:**
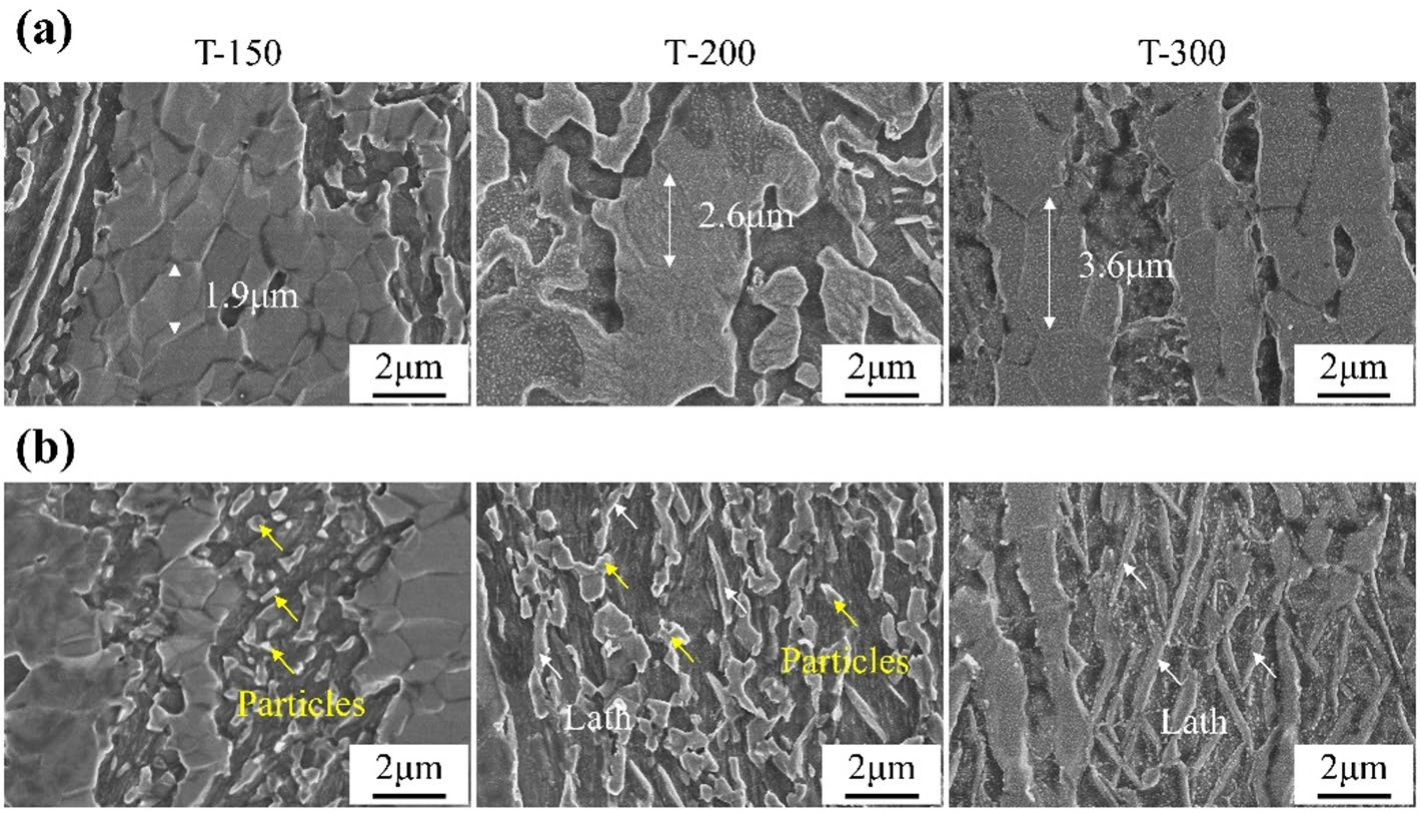
SEM morphologies of Zn-0.8Li alloys at different regions. **(a)** DRX grains. **(b)** Second phases.

Figure 2 highlights the DRX grains and secondary phases of the alloys. The microstructure consisted predominantly of fine recrystallized grains. For the T-150 sample, the average grain size was 1.9 μm. With higher ECAP temperatures, grain growth was evident, reaching 2.6 μm in T-200 and 3.6 μm in T-300. In terms of secondary phases, numerous fine particles were observed in T-150 and T-200, whereas the T-300 sample exhibited alternating lath-shaped Zn and LiZn₄ phases.

**Fig. 3:**
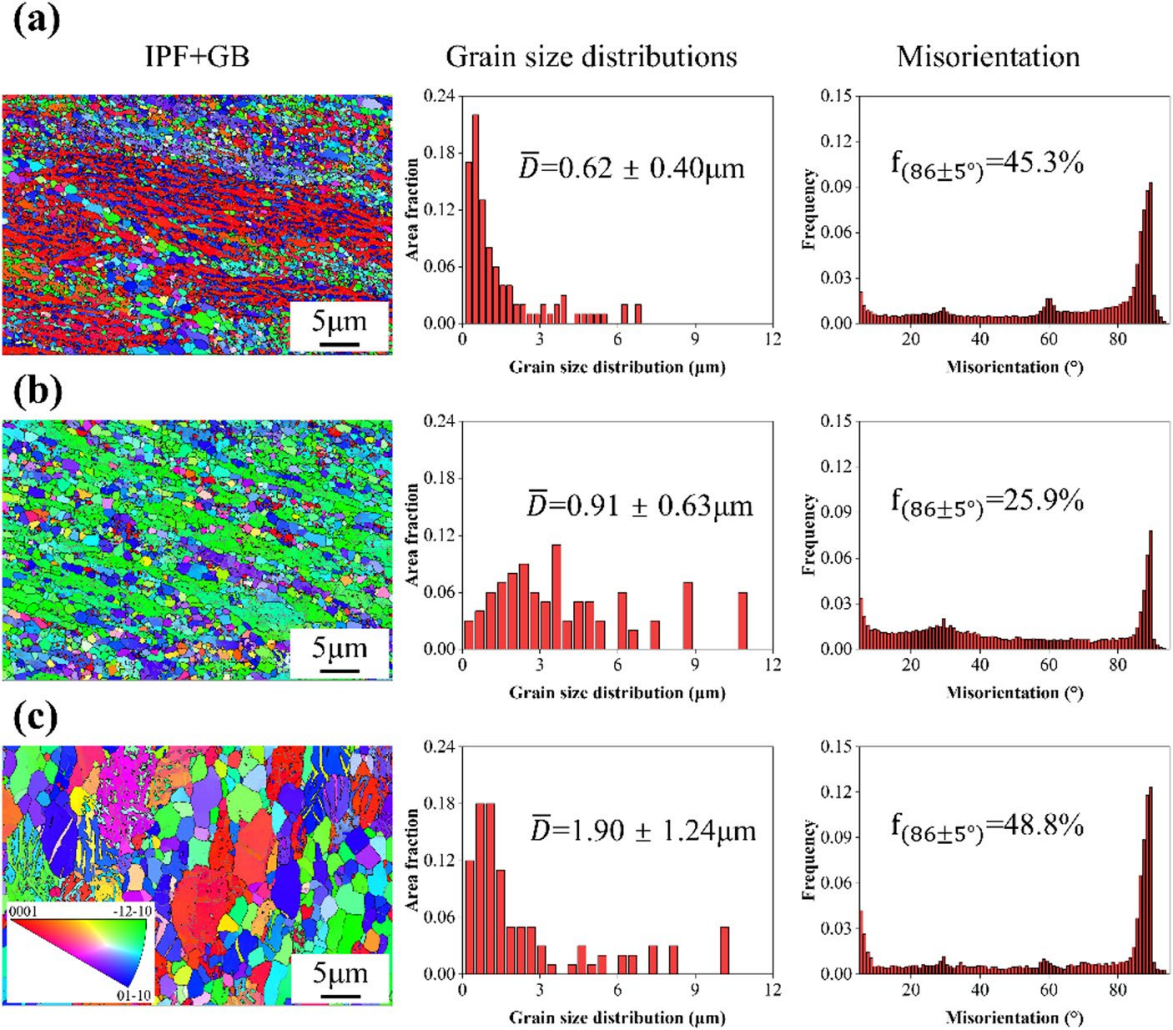
Grain size and misorientation distributions of Zn-0.8Li alloys. **(a)** T-150 sample. **(b)** T-200 sample. **(c)** T-300 sample.

EBSD analysis further revealed grain morphology and crystallographic orientation in Zn-0.8Li alloys subjected to ECAP (Fig. 3). In the inverse pole figure (IPF) maps, the gray regions corresponded to secondary phases, typically located along grain boundaries. Low-angle grain boundaries (LAGBs, 2°–15°) were marked as white lines, with fractions of 15.2%, 8.1%, and 12.4% for T-150, T-200, and T-300, respectively. Equiaxed ultrafine grains were evident across all conditions, confirming extensive dynamic recrystallization. The average grain sizes were 0.62 ± 0.40 μm for T-150, 0.91 ± 0.63 μm for T-200, and 1.90 ± 1.24 μm for T-300. In addition, twin boundaries were detected at misorientation angles of 86 ± 5°^24^. Overall, the evolution of grain size, boundary characteristics, and phase distribution clearly depended on ECAP processing temperature.

**Fig. 4:**
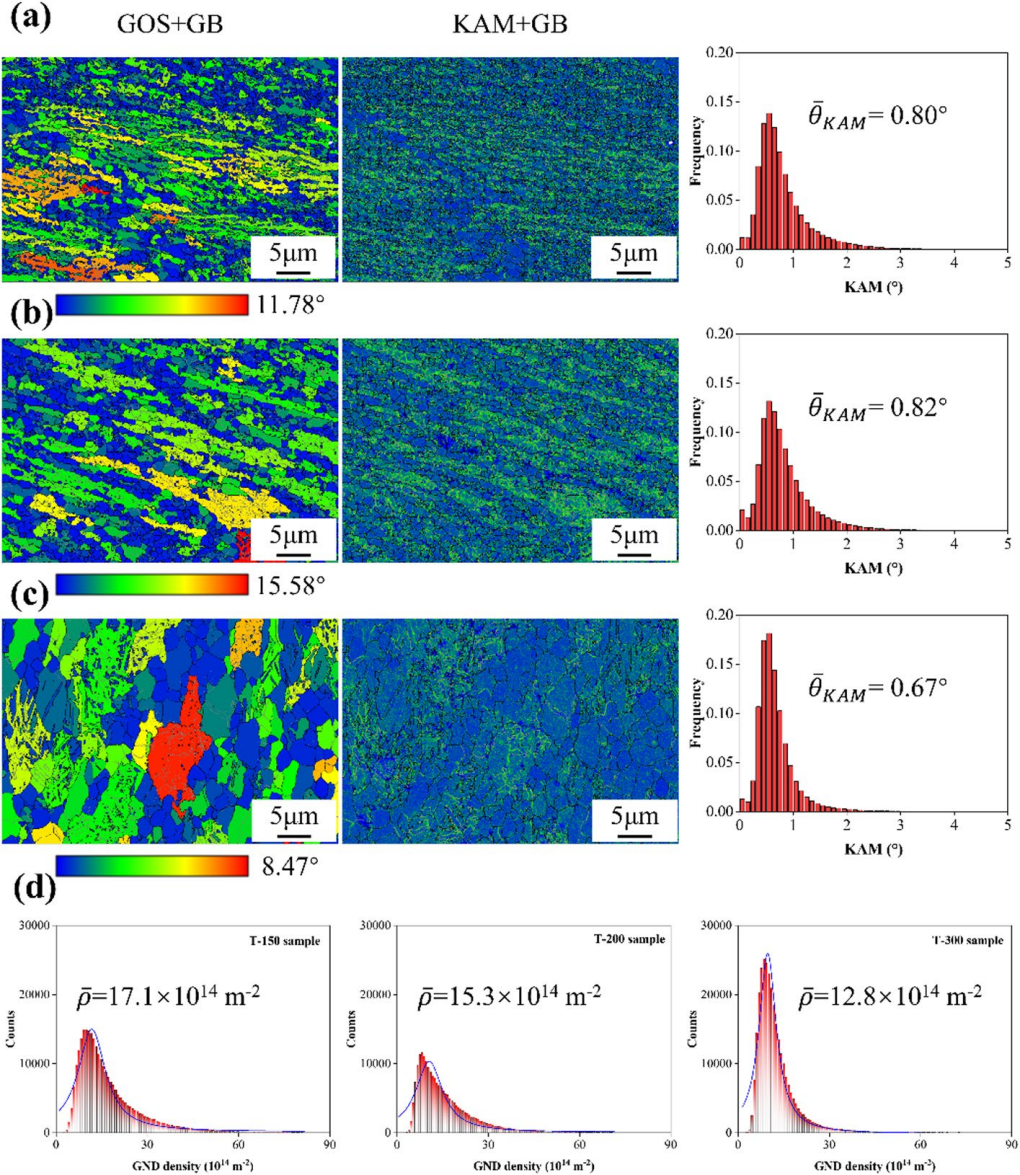
Recrystallization and dislocation density analysis of Zn-0.8Li alloys. **(a)** T-150 sample. **(b)** T-200 sample. **(c)** T-300 sample. **(d)** GND distributions for T-150, T-200, and T-300 samples.

Figure 4 shows the grain orientation spread (GOS) and kernel average misorientation (KAM) maps of the Zn-0.8Li alloys. In general, newly recrystallized grains display lower values, indicating the absence of deformation-induced substructures. The GOS results revealed that the fraction of recrystallized grains decreased slightly from 35.88% in T-150 to 34.65% in T-200, before increasing to 40.08% in T-300, suggesting that dynamic recrystallization (DRX) was promoted at higher ECAP temperatures. The average KAM angles were 0.80°, 0.82°, and 0.67° for T-150, T-200, and T-300, respectively. Since KAM values correlate with geometrically necessary dislocation (GND) density $\:\rho\:=\frac{2\stackrel{-}{\theta\:}}{\mu\:b}$, where ρ is dislocation density, θ is the mean KAM value, µ is the step size (0.07 μm), b the Burgers vector of Zn^25^. Figure 4d shows that the GND densities are 17.1 × 10¹⁴ m⁻², 15.3 × 10¹⁴ m⁻², and 12.8 × 10¹⁴ m⁻², showing a progressive reduction with rising processing temperature^26^.

**Fig. 5:**
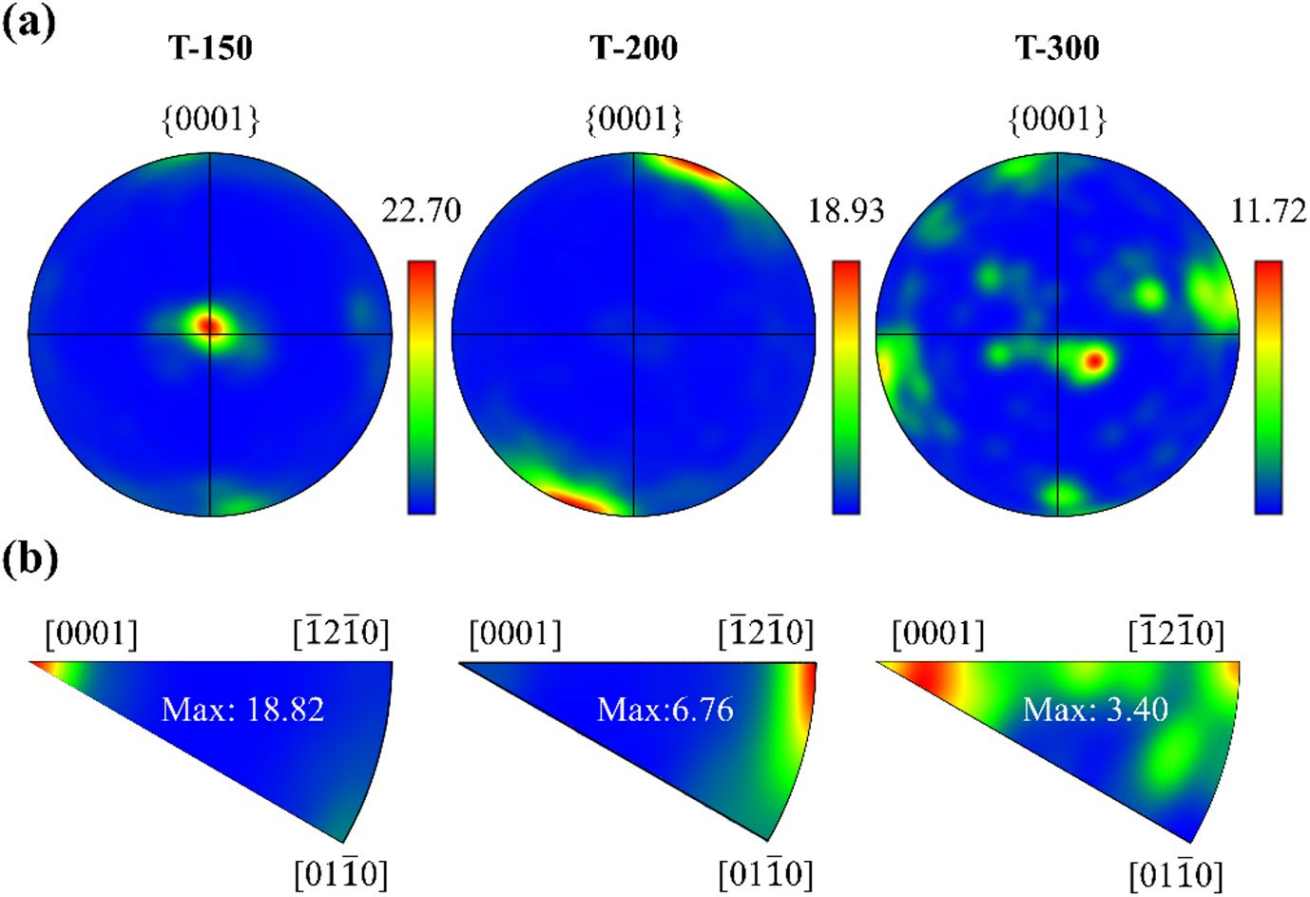
Texture analysis of Zn-0.8Li alloys. **(a)** Pole figures. **(b)** Inverse pole figures.

Pole figure (PF) mappings in Fig. 5a indicated that ECAP-processed Zn-0.8Li alloys exhibited a pronounced basal texture. The texture intensity weakened with higher deformation temperature, which can be attributed to the greater fraction of DRX grains and reduced dislocation density. To further elucidate textural evolution, inverse pole figure (IPF) maps were analyzed (Fig. 5b). At 150 °C, the alloy displayed a < 0001 > fiber texture. At 200 °C, the orientation shifted to <$\:\stackrel{-}{1}2\stackrel{-}{1}0$> orientation. At 300 °C, the texture reverted to <0001> fiber alignment. These results demonstrate a transformation of Zn-0.8Li alloys from basal fiber to non-basal orientations with increasing ECAP temperature, followed by re-establishment of the basal component at the highest temperature.

### Mechanical properties

**Fig. 6:**
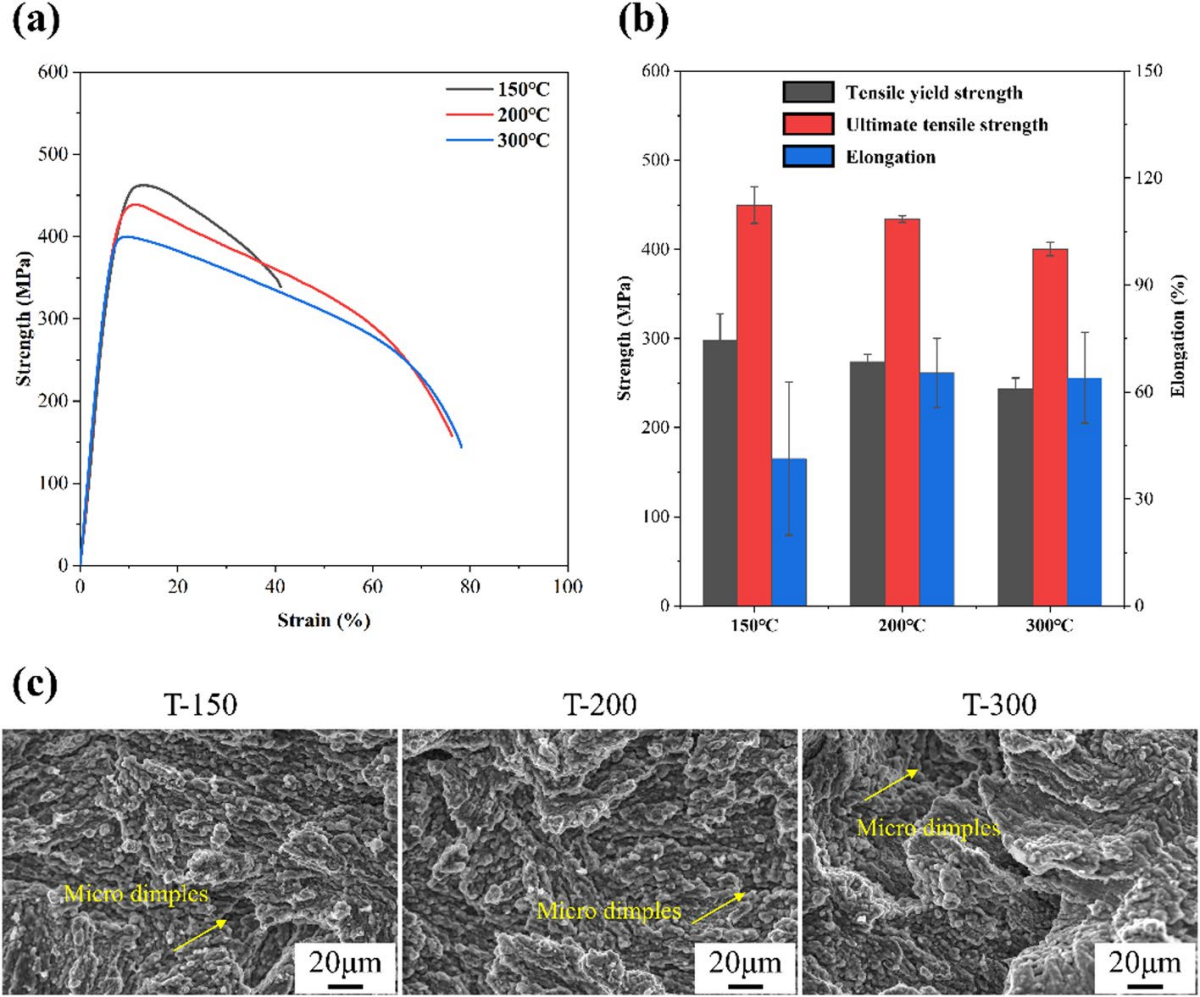
Mechanical properties of Zn-0.8Li alloys. **(a)** Engineering tensile curves. **(b)** Strengths and elongations. **(c)** Fractural morphologies.

The mechanical response of Zn-0.8Li alloys processed at different ECAP temperatures is summarized in Fig. 6. As shown in Fig. 6a, all three alloys exhibited elastic deformation, followed by mild strain hardening, reaching a peak stress and then softening until fracture. The evolution of tensile yield strength (TYS), ultimate tensile strength (UTS), and elongation with processing temperature is presented in Fig. 6b. Both TYS and UTS increased as ECAP temperature decreased. At 300 °C, the TYS and UTS were 244 ± 12 MPa and 400 ± 8 MPa, respectively. These values rose to 274 ± 1 MPa and 434 ± 4 MPa at 200 °C, and further to 298 ± 29 MPa and 450 ± 21 MPa at 150 °C. In contrast, ductility decreased from 64 ± 13% at 300 °C and 65 ± 9% at 200 °C to 41 ± 21% at 150 °C. Thus, low-temperature ECAP markedly enhanced strength but at the expense of elongation, underscoring the trade-off between strength and ductility in optimizing processing conditions.

The fracture surfaces are shown in Fig. 6c. For the T-300 sample, micro dimples and fine particles were observed, as shown by yellow arrows. Given the grain size was below 10 μm, cleavage planes were correspondingly small. The presence of numerous second phases and ~ 1 μm grains suggested that the fine particles originated from intergranular fracture or separation along phase boundaries. Similar features, including fine cleavage facets and particles, were also detected in the T-200 and T-150 samples. In addition, clear dimples were evident, especially at lower processing temperatures. Overall, increasing ECAP temperature shifted the fracture mode from predominantly brittle to a mixed brittle–ductile character, where microvoids and dimples helped accommodate plastic strain during tensile loading.

### *In-vitro* degradation behaviors

**Fig. 7:**
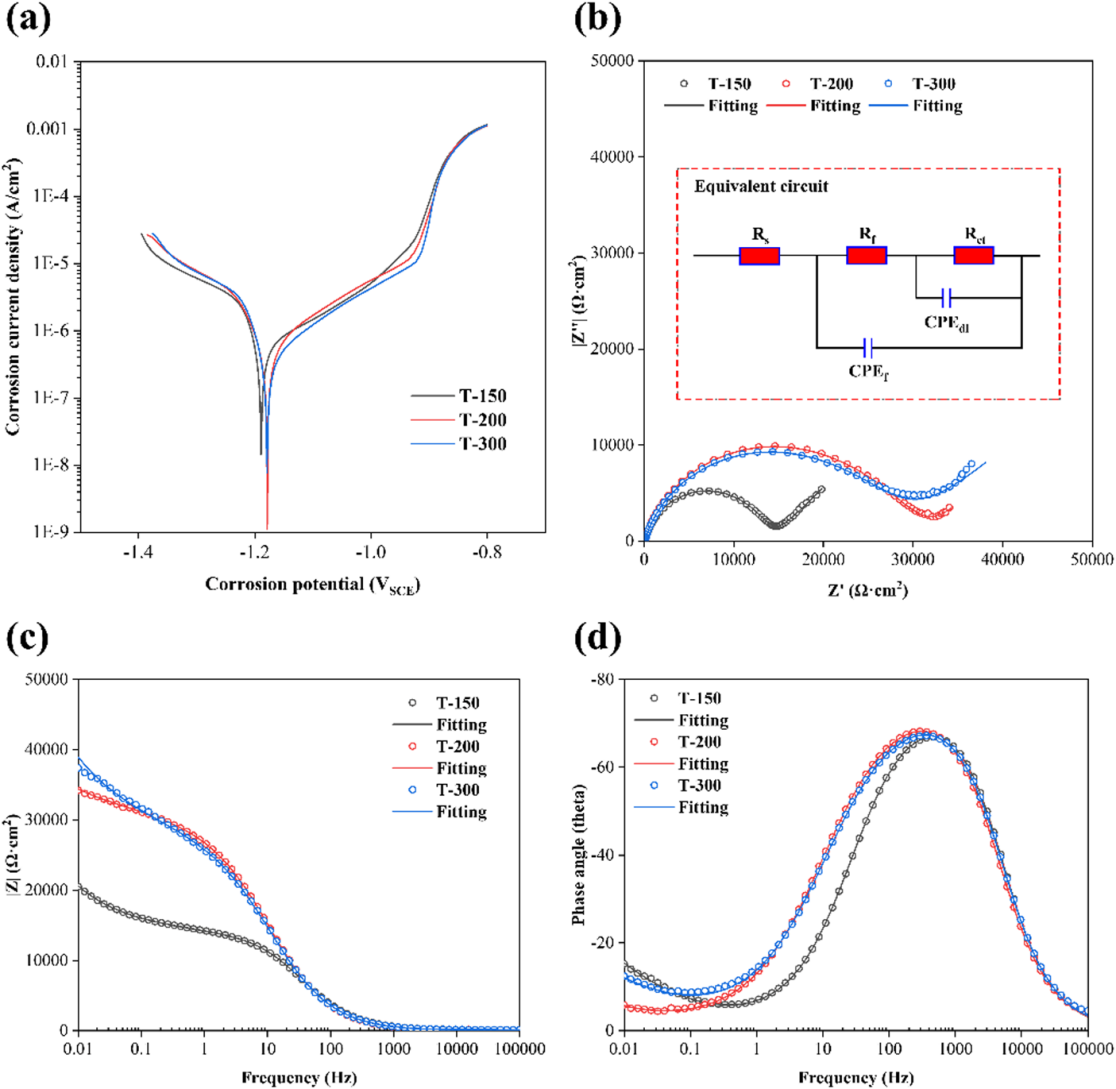
Electrochemical behaviors of Zn-0.8Li alloys. **(a)** PDP curves. **(b)** Nyquist plots and fitting curves. **(c)** Bode plots and fitting curves. **(d)** Phase angles and fitting curves.

Microstructural modifications induced by different ECAP temperatures not only improved strength but also had a pronounced effect on corrosion resistance. The electrochemical performance of Zn-0.8Li alloys in Hank’s solution is shown in Fig. 7. Potentiodynamic polarization (PDP) curves (Fig. 7a) revealed similar polarization processes across all samples. From these curves, corrosion potential and current density were extracted as key indicators of corrosion resistance. As summarized in Table 2, the alloys exhibited comparable corrosion potentials, but corrosion current density decreased progressively with increasing ECAP temperature, indicating improved anti-corrosion behavior. Nyquist plots (Fig. 7b) with the corresponding equivalent circuit model provided further insights. Fitting parameters are presented in Table 3. The diameter of the capacitive arc, which reflects polarization resistance, increased at higher processing temperatures. This trend was supported by rising values of R_ct_ and R_f_, implying that high-temperature ECAP promoted more protective surface films even though the corrosion product layers were thinner. Bode plots (Fig. 7c and d) confirmed these findings: both impedance modulus and phase angle increased systematically with temperature, suggesting enhanced stability and integrity of passive films. Collectively, the electrochemical analyses demonstrate that higher ECAP temperatures effectively suppress corrosion in Zn-0.8Li alloys by strengthening the protective capacity of surface layers.

**Table 2:** Corrosion potential and corrosion current density derived from PDP curves.

Samples	Corrosion potential (V vs. SCE)	Corrosion current density (A/cm^2^)
T-150	−1.19 ± 0.01	(7.16 ± 0.67) × 10^− 6^
T-200	−1.18 ± 0.02	(6.53 ± 0.72) × 10^− 6^
T-300	−1.18 ± 0.01	(6.49 ± 0.31) × 10^− 6^

**Table 3:** Fitting parameters in equivalent circuits.

Samples	*R*_s_ (Ω·cm^2^)	CPE_f_ (µF·cm^2^·s^n1^)	n1	*R*_f_ (Ω·cm^2^)	CPE_dl_ (µF·cm^2^·s^n2^)	n2	*R*_ct_ (Ω·cm^2^)
T-150	143.7	4.29	0.94	2208	1.92	0.62	11,886
T-200	142.5	4.71	0.91	2906	3.53	0.51	17,781
T-300	143.3	5.33	0.94	2869	3.12	0.54	17,462

**Fig. 8:**
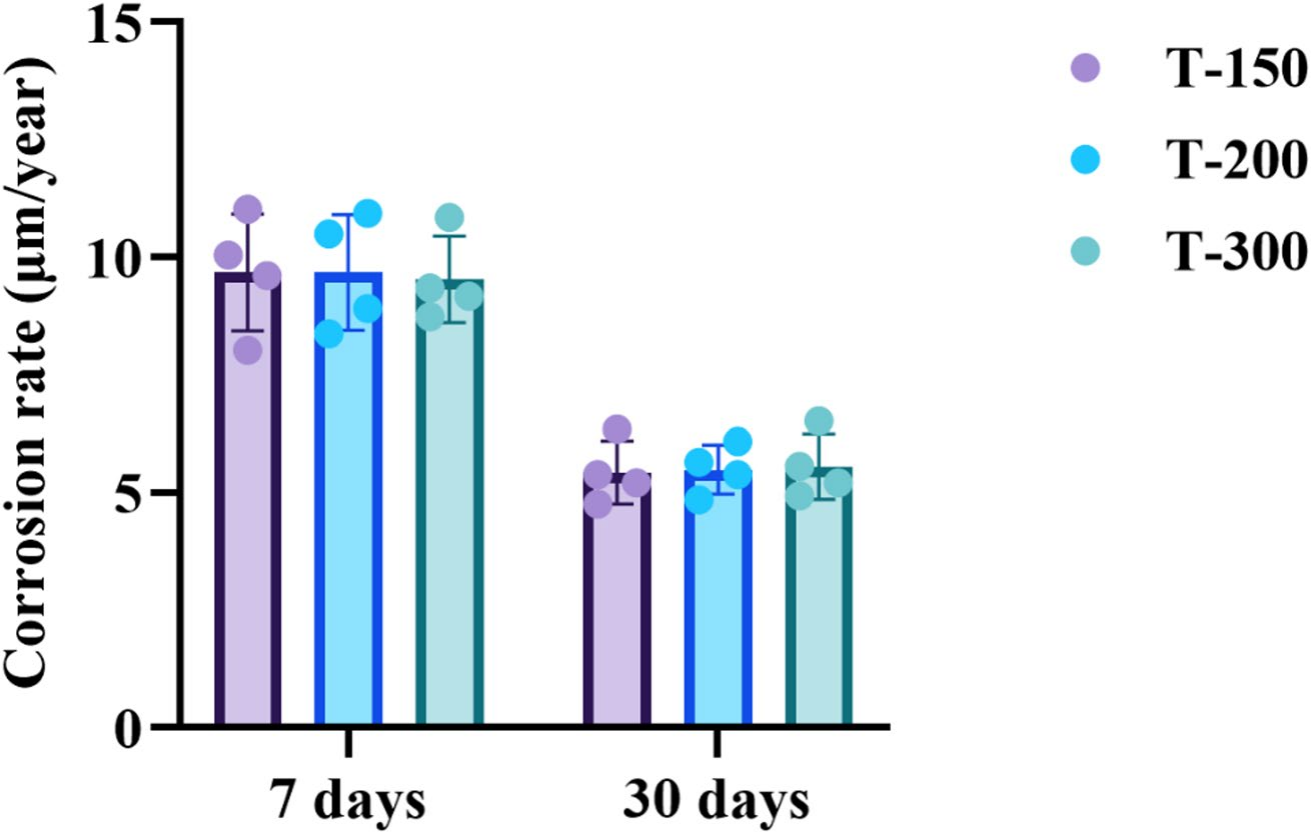
Corrosion rates of Zn-0.8Li alloys after ECAP at different temperature.

Corrosion rates derived from weight loss measurements after immersion are shown in Fig. 8. After 7 days in Hank’s solution, the Zn-0.8Li alloys exhibited rates of 9.67, 9.67, and 9.52 μm/year, respectively. After 30 days, all values dropped markedly compared with those at 7 days, reaching 5.42 μm/year for T-150, 5.49 μm/year for T-200, and 5.55 μm/year for T-300.

**Fig. 9:**
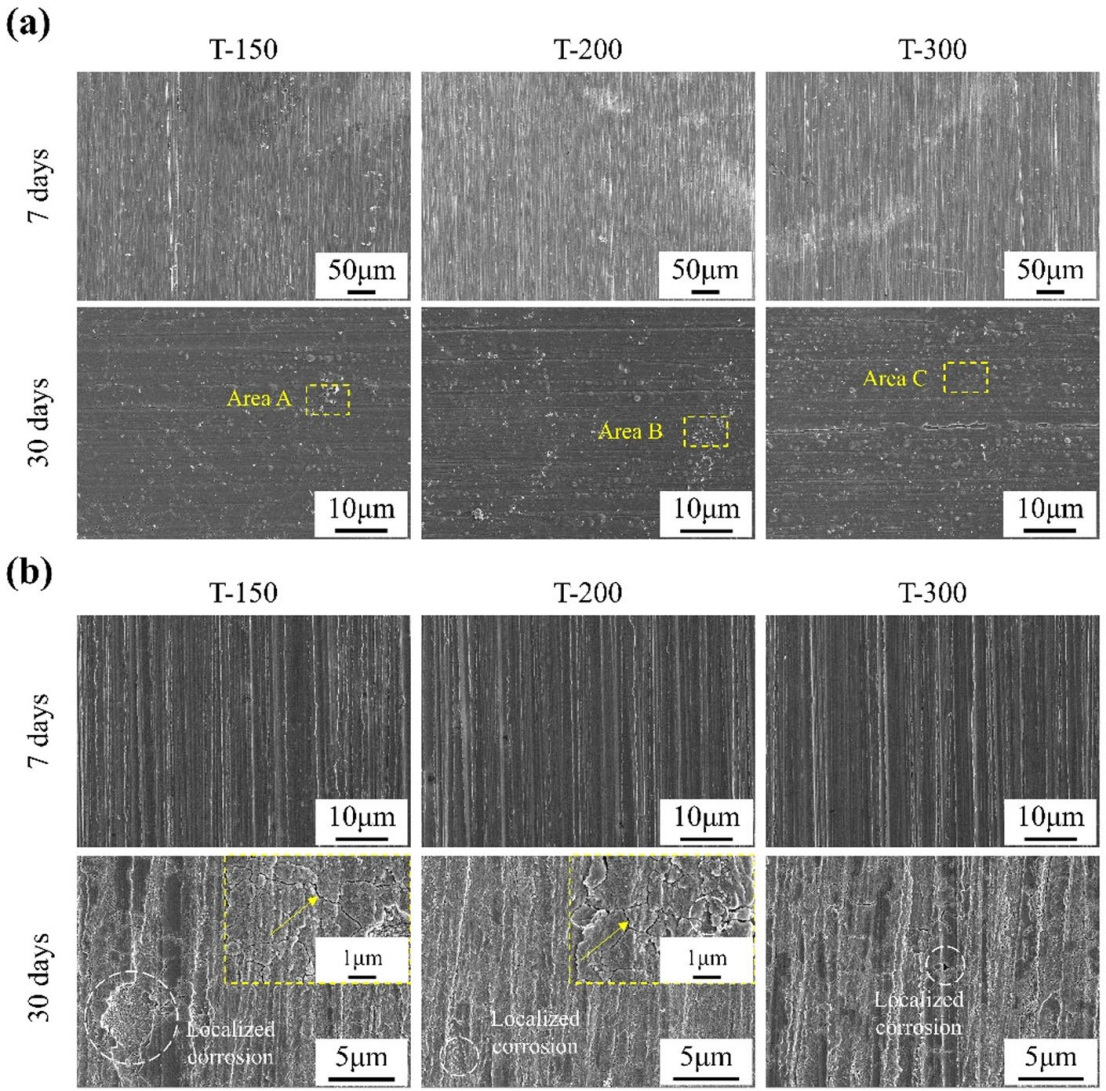
Surface morphologies of Zn-0.8Li alloys after immersion at different period. **(a)** SEM images before removing corrosion products. **(b)** SEM images after removing corrosion products.

The surface morphologies after 7 and 30 days of immersion are presented in Fig. 9. All samples were fully covered by corrosion product layers. After 30 days, numerous precipitated particles smaller than 2 μm were observed across all alloys. EDS analysis (Table 4) confirmed that corrosion products were mainly composed of carbon and oxygen, with minor amounts of calcium and phosphorus.

**Table 4:** Chemical compositions at different areas marked in Fig. 9a.

Areas	Weight fraction (wt%)				
	C	O	*P*	Ca	Zn
A	2.823	12.301	3.309	0.412	81.155
B	2.917	11.904	2.611	0.416	82.152
C	3.084	12.376	3.241	0.378	80.921

SEM images of the alloys after removal of corrosion products (Fig. 9b) revealed intact surfaces following 7 days of immersion. After 30 days, shallow pits appeared while the overall surfaces remained relatively flat. Both the number and size of pits decreased with increasing ECAP temperature. At higher magnification, intergranular cracks were observed along grain boundaries, together with strip-like localized corrosion features. The extent of localized damage was greater in T-150 samples, where pits exceeded 5 μm, but diminished significantly with higher processing temperatures, reaching below 1 μm in T-300.

### Cytocompatibility

**Fig. 10:**
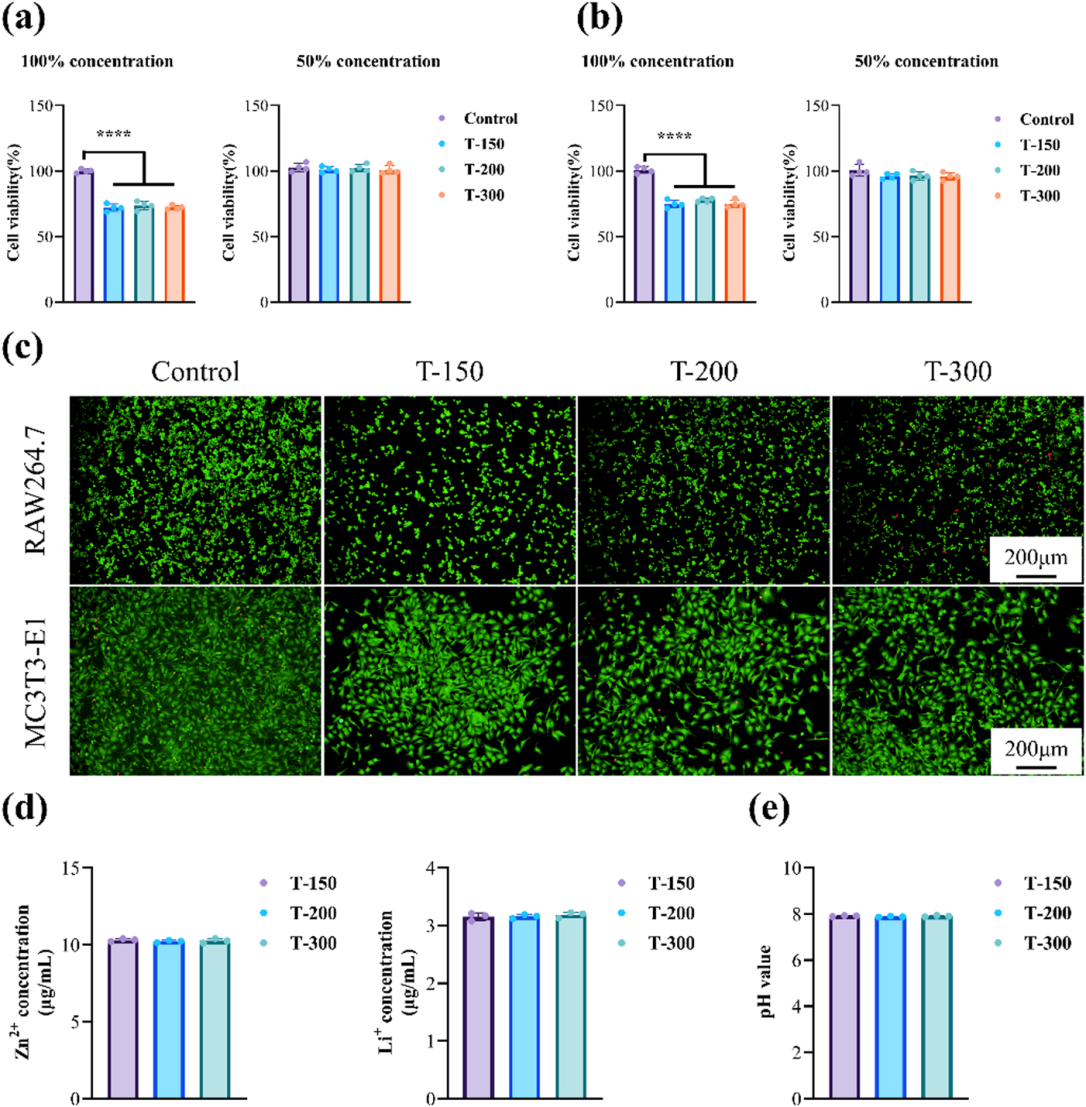
Cytocompatibility of the studied Zn alloys. Cell viability of **(a)** RAW264.7 **(b)** MC3T3-E1. **(c)** Live/dead staining images. **(d)** Ionic concentrations. **(e)** pH values of Zn alloys extracts.

The biocompatibility of Zn-0.8Li alloys was assessed using RAW264.7 and MC3T3-E1 cells relevant to osteogenesis, with inert titanium (Ti) as the control. Cell viability after 3 days of culture in different extract concentrations is shown in Fig. 10a and b. At 100% extract concentration, both cell types exhibited significantly lower activity compared with Ti, which can be attributed to elevated Zn²⁺ levels in undiluted extracts. When the concentration was reduced to 50%, Zn²⁺ levels decreased, leading to improved cell survival. Under these conditions, cell viability exceeded 90% after 3 days, demonstrating favorable cytocompatibility of the alloys. Live/dead staining (Fig. 10c) further confirmed high survival rates in cells exposed to alloy extracts. Measurements of ionic concentrations and pH in undiluted extracts (Fig. 10d and e) revealed no significant differences among the three Zn-0.8Li alloys, which explained the comparable cell viability results across groups.

### *In-vitro* osteogenesis

**Fig. 11:**
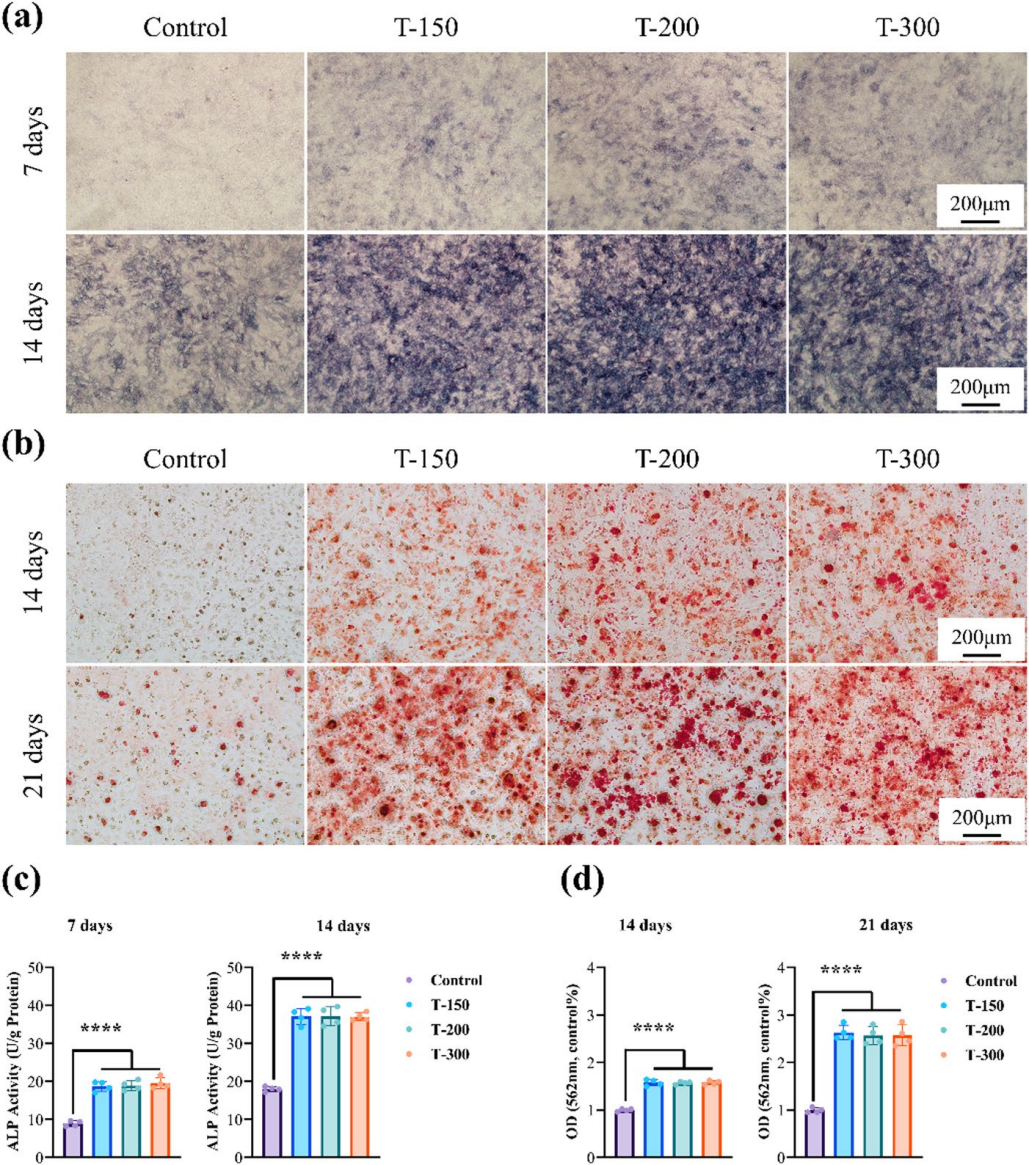
In-vitro osteogenesis of the studied Zn alloys. **(a)** ALP staining images. **(b)** ARS images. **(c)** ALP activity. **(d)** Absorbance of ARS.

To evaluate early osteogenic potential, ALP staining at 7 and 14 days and ARS staining at 14 and 21 days were performed on MC3T3-E1 cells cultured in 50% extracts (Fig. 11). Compared with Ti, Zn alloy groups displayed more intense ALP staining, with broader and darker purple regions (Fig. 11a). Calcified deposits also increased progressively in the alloy groups relative to the control (Fig. 11b). Quantitative analysis of ALP activity and ARS staining further confirmed higher osteogenic activity in Zn alloys than Ti, with ECAP-processed alloys outperforming cast ones. These findings demonstrate that Zn-0.8Li alloys exhibit strong early osteogenic induction, primarily due to the release of Zn²⁺ and Li⁺ ions, both of which are recognized as bone-promoting cations. Thus, the early osteogenic capacity of ECAP alloys benefits from the synergistic effect of degradation-derived ions.

### Antibacterial properties

**Fig. 12:**
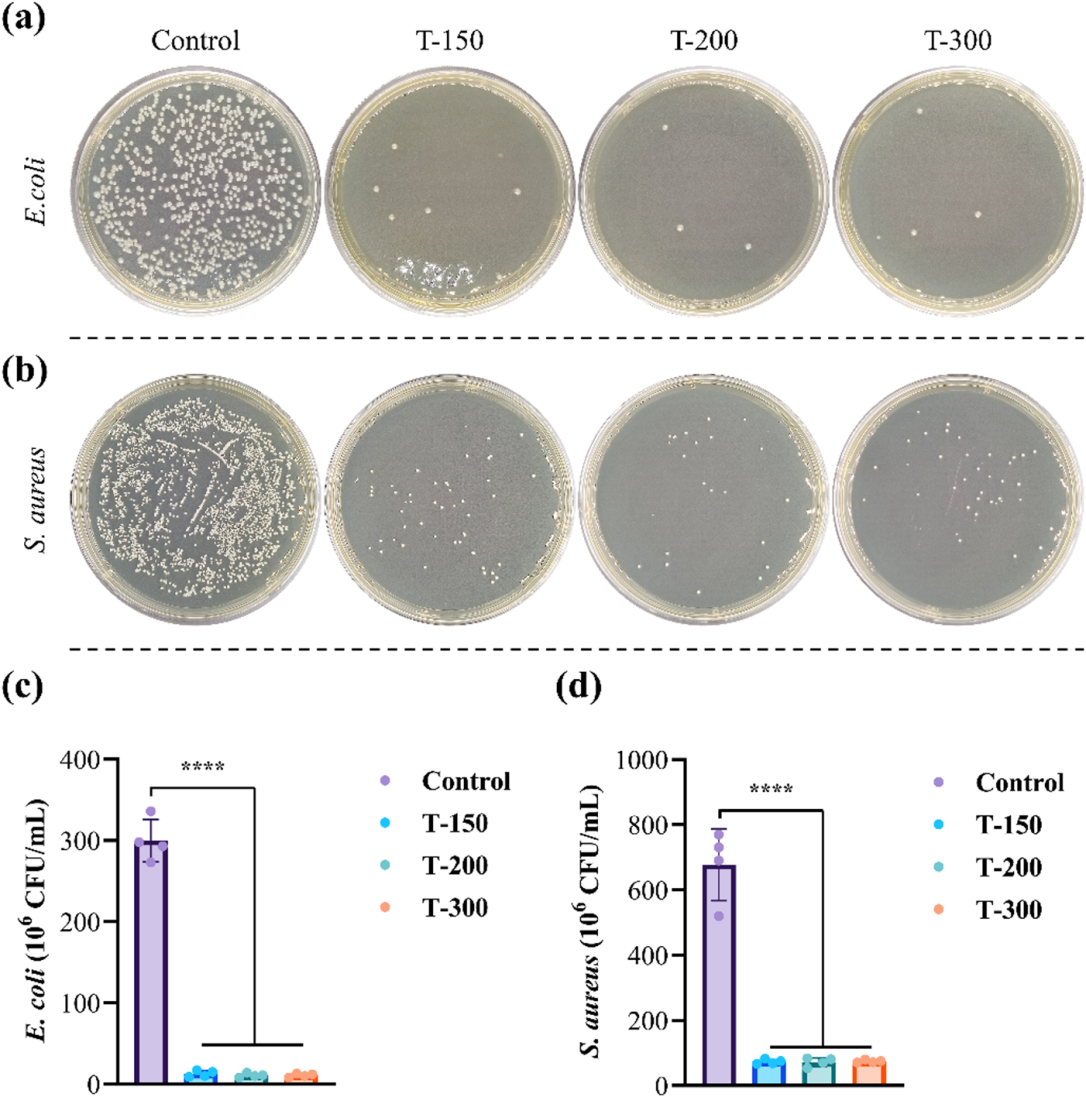
Antibacterial properties of the studied Zn alloys. CFU images of **(a)** *E.coli* and **(b)** *S.aureus* bacterial colonies. Quantitative analysis of antibacterial rate to **(c)** *E.coli* and **(d)** *S.aureus* bacterial colonies.

The antibacterial performance of the alloys against *S. aureus* and *E. coli* is shown in Fig. 12. After 24 h of incubation at 37 °C, significantly fewer bacterial colonies formed on Zn alloys compared with Ti. Against *S. aureus*, the antibacterial rates were 88.9 ± 2.3% (T-150), 88.9 ± 3.5% (T-200), and 88.8 ± 2.0% (T-300). For *E. coli*, the values were higher, reaching 95.6 ± 1.6% (T-150), 96.3 ± 1.0% (T-200), and 96.4 ± 0.8% (T-300). These consistently high rates confirm that ECAP Zn-0.8Li alloys exhibit robust antibacterial activity against both Gram-positive and Gram-negative bacteria.

## Discussions

### Effects of ECAP temperature on microstructure evolution

The microstructural evolution during ECAP is a decisive factor for the final architecture and overall performance of Zn–Li alloys. Our results confirm that lowering ECAP temperature effectively refined grains to the ultrafine regime. Similar submicron grain refinement has been reported for Zn-0.8Li alloys subjected to rolling or draw.

ing^14,27^. However, the role of ECAP temperature in controlling the behavior of the LiZn₄ phase has seldom been addressed. In the present work, eutectoid regions exhibited notable differences depending on processing temperature. *Huang et al.* emphasized that thermal conditions strongly influence phase constitution because of α-to-β transformations^19^. According to the Zn–Li phase diagram, the eutectic reaction L → β-LiZn₄ + Zn occurs at 401 °C, and β-LiZn₄ can subsequently transform into Zn and α-LiZn₄ at ~ 65 °C^21,28^. It can thus be inferred that increasing ECAP temperature promoted the formation of β-LiZn₄.

During severe plastic deformation, both the Zn matrix and β-LiZn₄ phases deform, but the Zn matrix—with lower hardness (1.03 GPa vs. 2.17 GPa)—accommodates strain first^29^. This initiates a dislocation-driven continuous DRX process in Zn, which progressively hardens the matrix and triggers plastic deformation of β-LiZn₄. The morphology, dimensions, and distribution of β-LiZn₄ particles were altered in a manner comparable to CuZn₅ phases in ECAPed Zn–Cu alloys^17,30^. Consequently, ECAP-processed Zn–Li alloys consist of DRX-refined Zn grains interspersed with deformed β-LiZn₄. Yet β-LiZn₄ is metastable at ambient temperature and may transform into α-LiZn₄, producing lamellar eutectoid features that preserve structural characteristics from solidification. This explains the clear distinction between DRX regions and eutectoid domains in the present alloys. At lower ECAP temperatures, incomplete β-LiZn₄ formation left behind Zn and α-LiZn₄ phases with limited plasticity. Hence, refinement mechanisms included both dislocation-mediated DRX and mechanical fragmentation of eutectoid lamellae. This refinement route resembles that of Zn–Mg alloys with brittle eutectic phases^31^. Fragmented eutectoid particles also hindered grain coarsening, contributing to the ultrafine grain structures observed after low-temperature ECAP. These results demonstrate that lowering ECAP temperature is an effective strategy to break down second phases by imposing sufficient equivalent strain. Since coarse, hard phases are often detrimental to strength and difficult to refine through conventional deformation, low-temperature ECAP provides a viable pathway to achieve ultrafine-grained Zn–Li alloys.

### Effects of ECAP temperature on mechanical properties

Tensile testing confirmed that the ultimate tensile strengths (UTS) of ECAP Zn-0.8Li alloys exceeded 300 MPa, fulfilling the mechanical requirements for orthopedic implants^6,32^. Microstructural analysis revealed that lowering ECAP temperature refined grains to the submicron scale and increased dislocation density, both of which directly influenced mechanical behavior. Thus, the observed strengthening can be rationalized by three classical mechanisms: grain boundary strengthening (σ_GB_), dislocation strengthening (σρ), and second-phase strengthening. Average grain sizes decreased from 1.90 μm (T-300) to 0.91 μm (T-200) and 0.62 μm (T-150), as lower processing temperature suppressed grain growth. Second-phase particles also stimulated dynamic recrystallization (DRX) through particle-stimulated nucleation (PSN), generating ultrafine DRX grains^33^. Conversely, higher processing temperatures facilitated boundary migration, partially counteracting refinement.

Grain boundaries act as effective barriers to dislocation motion, and the Hall–Petch relation ($\:{\sigma\:}_{y}={\sigma\:}_{0}+k{d}^{-\frac{1}{2}}$,)^34,35^ describes this size effect, where σy = 23 MPa for pure Zn, d is average grain size (µm), and k ≈ 0.22 MPa·m¹/² for deformed Zn alloys^36–38^. Calculated σ_GB_ contributions were 279 MPa (T-150), 230 MPa (T-200), and 160 MPa (T-300), underscoring the significant role of grain refinement in strengthening. Dislocation strengthening (σρ) arises from deformation-generated dislocations that hinder further slip. It can be expressed as $\:{\sigma\:}_{y}=M\alpha\:Gb\sqrt{\rho\:}$, where M = 2.1, α = 0.5, G = 43 GPa, and b = 0.267 nm^39^. Second-phase strengthening originates from lattice mismatch between the Zn matrix and LiZn₄ precipitates. Because Li has limited solubility, volume fractions of second phases were comparable across samples; however, particle size varied with processing temperature. At lower temperatures, finer particles formed, contributing to more effective strengthening. As shown in Fig. 5, the ECAP temperature greatly influences the textures of Zn-0.8Li alloys, showing a strong basal texture in T-150 sample. The effect of texture on YS can be explained by the following equation: $\:{\sigma\:}_{y}=(\frac{0.3}{m}-1)\times\:k{d}^{-0.5}$. Where m is the average Schmid factor value of basal slip^40^. The m values are 0.32, 0.31, and 0.22 for T-150, T-200, and T-300 samples, respectively. The calculated strength contributions from texture are − 16 MPa for T-150 sample, −7 MPa for T-200 sample, and 53 MPa for T-300 sample. Collectively, these results demonstrate that reducing ECAP temperature promotes ultrafine grains, higher dislocation density, and refined second-phase particles, all of which synergistically enhance the tensile strength of Zn-0.8Li alloys.

### Effects of ECAP temperature on corrosion behavior

The corrosion performance of ECAP Zn-0.8Li alloys was strongly dependent on the microstructures shaped by processing temperature. Previous studies have demonstrated that the corrosion rates of biomedical Mg alloys are controlled by microstructural features, including grain size and secondary phases^41–43^. In our study, the ECAP-processed Zn-0.8Li alloys displayed a bimodal structure consisting of dynamically recrystallized (DRX) grains interspersed with eutectoid regions, where Li was mainly present in the α-LiZn₄ phase. Given the much lower standard electrode potential of Li (− 3.04 V) compared with Zn (− 0.44 V), α-LiZn₄ is inherently more prone to corrosion^44^. Consequently, distinct corrosion modes were observed: DRX grain regions showed isolated pits near boundaries, whereas eutectoid domains exhibited localized attack caused by galvanic coupling between α-LiZn₄ and Zn.

In DRX regions, corrosion progressed primarily along grain boundaries, forming extended intergranular cracks. This mode is likely associated with micro-galvanic cells between grain boundaries and adjacent grain interiors, a phenomenon also reported in ultrafine-grained Zn alloys^20,45^. Differences among ECAP conditions were attributed to variations in grain size and boundary density. The measured line densities of high-angle grain boundaries (HAGBs) were 3.54 × 10⁶/m (T-150), 2.31 × 10⁶/m (T-200), and 1.43 × 10⁶/m (T-300). Higher boundary density reduces inter-boundary spacing, accelerates solute diffusion, and enhances chemical heterogeneity at boundaries, thereby intensifying intergranular corrosion. As a result, T-150 displayed long boundary cracks, whereas T-300 mainly exhibited discrete corrosion pits. The effect of grain size on corrosion behavior follows an inverse relation to the square root of grain size^46^. Grain refinement increases boundary density, which governs the conduction rate of surface oxide films. When the oxide layer is protective, smaller grains improve corrosion resistance. However, in Zn-0.8Li alloys, higher boundary density enhanced susceptibility to intergranular attack, despite the corrosion current density remaining below 10 µA/cm². Overall, these findings indicate that reducing ECAP temperature, while refining grains, exacerbates grain boundary corrosion due to intensified boundary reactivity, even though the alloys maintain relatively low overall corrosion currents.

### High-strength Zn–0.8Li alloys with enhanced osteogenic and antibacterial performance

In this study, ECAP-processed Zn-0.8Li alloys simultaneously released Zn²⁺ and Li⁺ ions during degradation (Fig. 10d). After one-fold dilution, the alloy extracts exhibited excellent cytocompatibility toward RAW264.7 and MC3T3-E1 cells (Fig. 10). Notably, the co-release of Li⁺ and Zn²⁺ significantly promoted in vitro osteogenesis, as evidenced by ALP and ARS staining. These findings suggest that degradation products of Zn-0.8Li alloys exert stronger osteogenic activity than pure Zn. Previous studies have provided insights into the underlying mechanisms about the effects of Zn^2+^ ions^11^. The PI3K-AKT pathway, a well-known regulator of bone repair, appears central to the osteogenic response. This pathway has been reported to mediate osteogenic differentiation in contexts such as microRNA and exosome-based bone regeneration^47^. In Zn–0.8Li-based alloys, Zhang et al. reported significantly higher expression of osteogenesis-related genes (ALP, COL1, OCN, and Runx2) compared with pure Ti ^10^. In addition to PI3K-AKT activation, Zn-binding metallothioneins are also elevated, contributing to intracellular Zn homeostasis and promoting bone formation^48^. Beyond osteogenic performance, ECAP-processed Zn–Li alloys showed antibacterial rates exceeding 90% against *S. aureus* and *E. coli* (Fig. 12). This activity is primarily attributed to the broad-spectrum antibacterial effects of Zn²⁺^49^, which can increase intracellular reactive oxygen species (ROS), disrupt bacterial structures, impair metabolism and DNA replication, and inhibit proliferation^50^. Notably, Zn²⁺ exhibits dose-dependent antibacterial effects, with attenuated activity reported above 12 µg/mL^51^. In the present study, ICP results indicated Zn²⁺ and Li⁺ concentrations of approximately 10.3 µg/mL and 3.2 µg/mL, respectively, suggesting that co-release of Zn²⁺ and Li⁺ may contribute to the observed antibacterial efficacy. This multifaceted response highlights the alloys’ promise as bioactive materials capable of combining mechanical strength with superior bone-regeneration and antibacterial potential.

## Conclusions

This work systematically examined the microstructure, mechanical performance, corrosion response, and biocompatibility of Zn–Li alloys subjected to ECAP at different temperatures. The main findings are summarized as follows:

(1) ECAP processing refined the microstructure significantly. The average grain size reached 0.62 μm for T-150, 0.91 μm for T-200, and 1.90 μm for T-300. Grain refinement was mainly driven by dynamic recrystallization. At low ECAP temperatures, incomplete transformation of α-LiZn₄ into β-LiZn₄ produced fragmented secondary phases. At higher temperatures, deformed β-LiZn₄ became unstable and eventually transformed into α-LiZn₄ and Zn.

(2) The processed Zn–Li alloys exhibited tensile strengths exceeding 400 MPa, satisfying the mechanical criteria for orthopedic implants. However, ductility decreased from 65% (T-300) to 41% (T-150) as temperature decreased. Strengthening was dominated by grain boundary and dislocation mechanisms.

(3) Corrosion behavior was strongly dependent on temperature. Localized attack occurred in secondary phases, with intergranular corrosion prominent in T-150 and T-200, while boundary attack was suppressed in T-300. Despite this, all alloys maintained corrosion current densities below 10 µA/cm². Biologically, ECAP Zn–Li alloys demonstrated excellent cytocompatibility, promoted osteogenic activity, and achieved antibacterial rates above 90% against *E. coli*.

Overall, these findings highlight ECAP as an effective strategy to tailor Zn–Li alloys with ultrafine-grained microstructures, high strength, and promising biological functions for next-generation biodegradable orthopedic implants.

## Data Availability

The datasets used and analysed during the current study available from the corresponding author on reasonable request.
